# Characterization and Comparison of Corrosion Layer Microstructure between Cement Mortar and Alkali-Activated Fly Ash/Slag Mortar Exposed to Sulfuric Acid and Acetic Acid

**DOI:** 10.3390/ma15041527

**Published:** 2022-02-18

**Authors:** Wenjing Zhao, Zirui Fan, Xin Li, Lijuan Kong, Liying Zhang

**Affiliations:** 1 School of Materials Science and Engineering, Shijiazhuang Tiedao University, Shijiazhuang 050043, China; 1202008069@student.stdu.edu.cn (W.Z.); fanzirui@stdu.edu.cn (Z.F.); luokunzhang@stdu.edu.cn (X.L.); 2 State Key Laboratory of Mechanical Behavior and System Safety of Traffic Engineering Structures, Shijiazhuang Tiedao University, Shijiazhuang 050043, China; 3 Shanghai Collaborative Innovation Center for High Performance Fiber Composites, Center for Civil Aviation Composites, Donghua University, Shanghai 201620, China; lyzhang@dhu.edu.cn

**Keywords:** alkali-activated mortar, acid attack, corrosion layer, microstructure, alternating current impedance

## Abstract

In this study, we investigated the formation and evolution of the corrosion layers in alkali-activated mortar and ordinary Portland cement mortar exposed to sulfuric acid and acetic acid environments with different pH values, and explored the differences in the deterioration mechanisms. The experimental results indicated that ordinary Portland cement (OPC) mortars experienced more severe deterioration in terms of appearance, mass loss, and strength loss as compared with alkali-activated mortars exposed to an acetic acid environment, but their neutralization depths were smaller. Alkali-activated fly ash (AAF) mortar had a the relatively intact appearance but the greatest neutralization depth, which was due to its stable three-dimensional network but highly porous structure. To sum up, alkali-activated fly ash/slag (AFS) mortar had the best resistance to acid attack. In addition, the mortars exposed to acetic acid suffered greater deterioration than those exposed to sulfuric acid with the same pH values, which was mainly due to the highly porous corrosion layer formed in acetic acid, whereas crystallization of gypsum in sulfuric acid had a pore filling effect. However, for alkali-activated slag (AAS) and OPC mortars exposed to a sulfuric acid environment, extensive gypsum resulted in the formation of micro-cracks, and the corrosion layer of OPC mortar was more prone to fall off. OPC mortar also had the greatest resistance difference values of the continuously connected micro-pores before and after acid corrosion, followed by AAS, AAF, and AFS mortars, and these values for all the specimens were smaller in sulfuric acid. Furthermore, the gaps between acetic and sulfuric acid attacks increased with increased calcium content in binders, which were 7%, 13%, 21%, and 29% for AAF, AFS, AAS, and OPC mortars, respectively. Thus, it can be inferred that an appropriate amount of gypsum existed in the corrosion layer which could act as a barrier to some extent ina sulfuric acid environment.

## 1. Introduction

Underground sewer networks are part of urban lifeline projects. At present, the entire world is faced with the challenge of aging and deteriorating sewage pipelines, and it is expensive to maintain and renew concrete sewers [1]; therefore, there is an urgent need to find concrete materials with better durability. In the service process of concrete sewer pipes, microbial effect [2] is considered to be the main cause of concrete degradation in sewage environments, apart from physical and chemical effects [3,4]. This is because in sewage there are high numbers of organic and inorganic substances containing carbon, hydrogen, oxygen, nitrogen, sulfur, and other elements, which are eventually decomposed into carbon dioxide, sulfate, nitrate, hydrogen sulfide, etc., under the metabolic action of microorganisms, and further transformed into sulfuric acid, nitric acid, and various organic acids [5,6,7]. Among the acids, biological sulfuric acid produced by sulfur oxidizing bacteria (SOB) under aerobic conditions is the main cause of concrete degradation in the unfilled space of gravity sewage pipes [8], which often originates from the sewage level and gradually develops to the crown of the pipe [9].

Alkali-activated binder is a new type of cementitious material [10], which is prepared with natural minerals or solid wastes rich in active silicate/aluminate as raw materials and under the action of an alkali activator. It is green and environmentally friendly, and also has better mechanical properties and durability than traditional cement [11]. Numerous studies in the literature have reported that the resistance of alkali-activated concrete to acid attack is better than that of ordinary concrete [12,13]. For example, Kwasny et al. [14] compared the sulfate and acid resistance of lithomarge-based geopolymer mortars and Portland cement mortars. A better performance was observed for the geopolymer mortar, which had no obvious deterioration in appearance and low mass loss. In particular, the geopolymer mortars prepared with sodium hydroxide as the activator had a more stable structure in an acidic environment [15]. Xie et al. [16] studied the corrosion resistance of ordinary Portland concrete and alkali-activated concrete subjected to biogenic sulfuric acid attack, and found that the latter had better performance in terms of appearance, mass loss, and strength deterioration. Furthermore, biological sulfuric acid was more corrosive to geopolymer concrete than chemical sulfuric acid, however, their corrosion mechanisms were the same and the main products of both were gypsum [17]. Bernal et al. [18] found that the appearance of alkali-activated slag mortar had almost no change after exposure to acetic acid, while the strength and pore structure of the cement mortar were seriously degraded.

The deterioration mechanism of a sulfuric acid attack on ordinary concrete is due to the decomposition of C-S-H gels and generation of expansive gypsum and ettringite [19], whereas an organic acid attack is due to the continuous decomposition and dissolution of hydrated products [20]. The good acid resistance of geopolymer concrete mainly comes from its relatively stable three-dimensional network structure. However, skeletal charge compensating cations (Na^+^ and Ca^2+^) undergo ion-exchange reactions with H^+^ or H_3_O^+^ in an acidic environment, and the electrophilic attack of acid protons on the Si-O-Al bond of geopolymer concrete leads to the discharge of aluminum from the aluminosilicate skeleton and to the deterioration of pore structure [21]. In a sulfuric acid solution with high concentration, the Ca^2+^ cations in geopolymer concrete react with the reverse diffused SO_4_^2−^ to form gypsum crystals in the corrosion layer [22], whereas there is no gypsum found in the corrosion layer of geopolymer concrete exposed to sulfuric acid with a pH value of three, which is similar to that of nitric acid [21]. Certainly, the amount of gypsum is also associated with the calcium content in silicate/aluminate raw materials. Regarding fly ash-based geopolymer mortar with a low calcium content, the structure is dominated by N-A-S-H gel, whereas the structure of alkali-activated granulated blast furnace slag (GBFS) mortar with a higher calcium content is mainly C-S-H and C-A-S-H gels [23]. After one-year exposure in field sewer pipelines, the neutralization depths of alkali-activated fly ash and slag mortars have been reported to be 25.5 mm and 5.2 mm, respectively [24]. Moreover, the strength loss rates of fly ash and GBFS geopolymer concrete and ordinary Portland cement concrete after 9 months of immersion in 3% sulfuric acid solution were 10.9%, 7.3%, and 26.6%, respectively [25]. Obviously, alkali-activated slag paste had better impermeability and acid resistance [26], which was due to generation of C-(A)-S-H gel with a finer pore structure [27]. However, the neutralization depth of fly ash-based geopolymer mortar was larger than that of sulphate resistant Portland cement mortar exposed to an acidic environment, which was due to the deterioration of the pore structure by the dealumination of N-A-S-H gel [28].

Overall, alkali-activated concrete after acid corrosion has a relatively intact appearance without obvious peeling and a small mass loss but larger corrosion depth [29]. Regarding conventional concrete suffering sulfuric acid attack, the gypsum corrosion layer formed on its surface can block the pores and, to a certain degree, reduce the penetration of corrosion medium [30]. However, some studies have found that both the thickness and porosity of the corrosion layer formed on the surface of ordinary cement concrete after biological sulfuric acid exposure are significantly greater than that of geopolymer concrete [13]. Thus, it is clear that the product, microstructure, and depth of corrosion layer formed on the surface of materials all have important influences on the deterioration of concrete, which are related to the silicon-aluminum powder variety, the type and concentration of acidic solutions, and so on. Therefore, in this study, the formation and evolution of the corrosion layers in ordinary Portand cement mortar and alkali-activated fly ash/slag mortars exposed to sulfuric acid and acetic acid environments with different pH values were investigated, to further explore the differences in the deterioration mechanisms.

## 2. Experimental Procedure

### 2.1. Materials and Specimens

#### 2.1.1. Raw Materials

In this study, Class F fly ash and S95 GBFS were used as the raw materials for the preparation of alkali-activated mortar, and Grade 42.5 Ordinary Portland Cement was used to prepare the control mortar for comparison. Their chemical compositions are listed in Table 1. The alkali activator was prepared by blending sodium silicate solution and 10 M sodium hydroxide, which was used after aging for 24 h. The chemical composition of the sodium silicate solution by mass was Na_2_O = 8.38%, SiO_2_ = 26.41%, and water = 65.21%. The modulus of blended sodium silicate-based solution was 2.0. River sand with a fineness modulus of 2.6 was used as the fine aggregate.

#### 2.1.2. Specimen Preparation

The alkali-activated fly ash (AAF), alkali-activated slag (AFS), alkali-activated fly ash/slag (AAS), and ordinary Portland cement (OPC) mortar mixtures were designed with the same water/binder ratio of 0.5. The water-solid ratio was calculated based on the ratio of total water (added water, water in sodium silicate and sodium hydroxide) to all the solid materials (silicon-aluminum materials, sodium hydroxide and solids in sodium silicate). Table 2 shows the mix details for the mortars. After mixing, the fresh mortars were poured into 40 × 40 × 160 mm^3^ molds. First, they were steam cured at a temperature of 80 °C for 24 h, and then placed in a standard curing room (T = 20 °C, RH ≥ 95%) to a specified age.

### 2.2. Experimental Design

The mortar specimens were immersed in solutions of CH_3_COOH and H_2_SO_4_ with different pH levels, which were prepared by adding concentrated acid to distilled water. The pH values of acetic acid and sulfuric acid were controlled around the values of 1.0 (1A, 1S) and 3.0 (3A, 3S), respectively, by monitoring with a pH meter. The liquid level was maintained at 20 mm higher than the top surface of the mortar specimens throughout the entire tests. The pH values of the acidic solutions were measured every day, and the acidic solutions were replaced with fresh solutions every 7 days to maintain the designed acidic environments.

### 2.3. Test Methods

#### 2.3.1. Appearance and Neutralization Depth Observation

After 28 days of immersion in acidic solutions, the mortar specimens were removed and washed with clean water, and then their appearance was observed visually. Moreover, the specimens were split and 1% phenolphthalein was sprayed on their fracture surfaces. The neutralization depths of specimens were determined according to the color of the sections, which followed the rule that a purple colored area indicated “unaffected by acid attack”, whereas a no color area indicated that neutralization occurred in the mortar.

#### 2.3.2. Mass and Strength Test

The mass of mortar specimens before and after acid immersion were measured by using a digital balance with a precision of ±0.01. Before weighing, the specimens were washed with clean water to remove the soft corrosion layer, and the saturation surface was kept dry. The specimens (40 × 40 × 160 mm^3^ in dimension) were used to test the strength according to the Chinese standards of GB/T 50081-2002. First, a three-point bending test was conducted to determine the flexural strength of the specimens. Then, the semi prism specimens after fracture were tested to determine the compressive strength. 

#### 2.3.3. X-ray Diffraction (XRD) and Scanning Electron Microscope (SEM)-Energy Dispersive Spectrometer (EDS) Analysis

The mineral components of the corrosion layers of the specimens were tested by Bruker D8 XRD (Karlsruhe, Germany) after exposure to acidic solutions. The spectra used were in the range of 5° < 2θ < 90°. Furthermore, the micromorphology and element distributions of the specimens were examined by a HITACHI S4700 SEM (Tokyo, Japan) equipped with EDS. Before testing, a Balzer sputtering coater (Tokyo, Japan) was used to apply a layer of gold coating on the specimens. 

#### 2.3.4. Alternating Current (AC) Impedance Test

In order to characterize the differences in the microstructures between alkali-activated mortars and cement mortar, as well as the evolution after being subjected to acid attack, the specimens were tested by AC impedance, which is a nondestructive method with high sensitivity. An IM3570 impedance analyzer (Nagano, Japan) was used, which measured impedance from 5 MH to 1000 Hz, and the amplitude of the sinusoidal voltage was chosen to be 10 mV. Before testing, the samples were vacuum water saturated, and filter papers, immersed in NaOH solution, were placed between the specimens and electrodes under pre-tightening force using clamps to ensure close contact.

## 3. Results and Discussion

### 3.1. Deterioration Behavior of Mortar Exposed to Acids

#### 3.1.1. Corrosion Morphology

**Figure 1:**
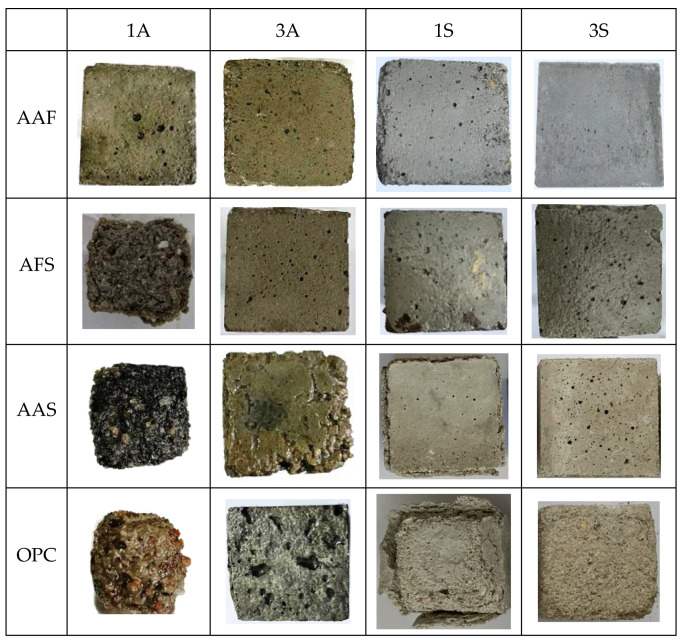
Appearance of mortar specimens after acid corrosion.

Figure 1 illustrates the appearance of the corroded mortar specimens after 28 days of immersion in acidic solutions. It can be observed that the mortar specimens immersed in acetic acid with a pH value of one suffered the most serious acid attack, and among them, the AAF mortar specimen showed a relatively intact appearance, only a few etch pits appeared on its surface, whereas the other specimens had obvious spalling; no prominent angularities could be observed, especially in the OPC mortar. In the sulfuric acid environment, the AAF mortar specimen also exhibited good resistance to acid attack, which may have been due to the 3D network structure of N-A-S-H gels which have higher stability. The acidic solutions with a pH value of three had a weak attack on the mortars. In addition, the acid resistance of alkali-activated mortars decreased with an increase in slag content. The surface of the AAS mortar specimen appeared to be very rough and some aggregates were exposed due to the dissolution and spalling of paste. Moreover, some white substance was observed on its surface after it was immersed in sulfuric acid with a pH value of one, which was inferred to be gypsum. Similarly, the OPC mortar also had a white appearing covering, due to a loose gypsum corrosion layer after sulfuric acid attack, and larger etch pits formed in the 1S solution.

#### 3.1.2. Mass Change

**Figure 2:**
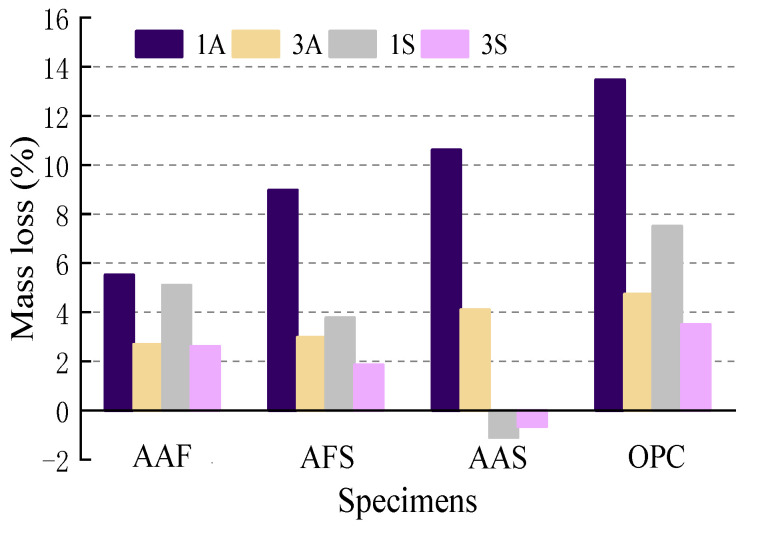
Mass loss of mortar specimens exposed to acid.

The mass change values of the mortars exposed to acidic solutions were determined by comparing the masses of the corroded specimens with the initial non-corroded specimens. The results are shown in Figure 2. A positive value means a decrease in mass, whereas a negative value means an increase in mass. It can be seen that the mass loss values of mortar specimens in the 1A solution were significantly higher than those in the other acidic solutions, which were 5.5%, 8.9%, 10.6%, and 13.4% for AAF, AFS, AAS, and OPC mortar specimens, respectively. This is consistent with the results observed in corrosion morphology. The OPC specimens also exhibited the highest mass loss values in other acidic environments, indicating more severe deterioration as compared with alkali-activated mortars, but the mass loss in sulfuric acid was lower than that in acetic acid with the same pH value. It can be inferred that the gypsum formed on the surface of cement mortar in a sulfuric acid environment fills the pores and holes and acts as a barrier layer, thereby reducing the penetration of the acidic solution. For alkali-activated mortars, the mass loss values increased with an increase in slag content in specimens exposed to acetic acid, but they demonstrated the opposite in sulfuric acid, the mass loss value of the AAS mortar specimens even slightly reduced after sulfuric acid immersion. This can be explained as follows: An increase in slag content of alkali-activated mortars led to the generation of more C-(A)-S-H gels, which were unstable in the acetic acid environment and easily decomposed and dissolved, thus, resulting in a higher mass loss of specimens. However, the C-(A)-S-H gels in the AFS and AAS mortar specimens reacted with the sulfuric acid and generated some gypsum, which reduced the mass loss of the mortars to a certain extent. Furthermore, the corrosion layer formed on AAS mortar was not easy to peel off as compared with OPC mortar, and together with the water absorption of specimens in acidic solution, they were the main causes of the reduction in mass loss after being exposed to sulfuric acid.

#### 3.1.3. Neutralization Depth

**Figure 3:**
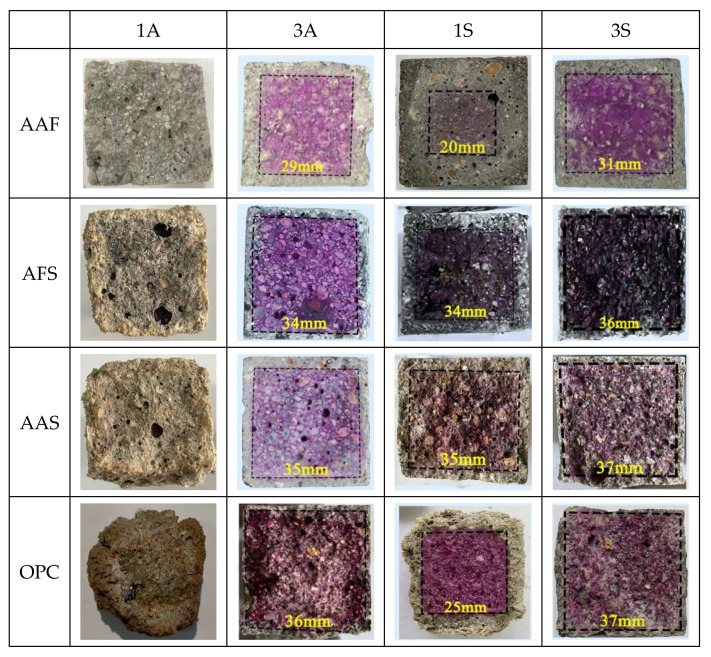
Fracture surface appearance of the corroded mortars after phenolphthalein spraying.

Figure 3 shows the fracture surface appearance of the corroded mortar specimens after phenolphthalein spraying. It can be seen that all the mortar specimens lost their alkalinity completely after 28 days of immersion in acetic acid solution with a pH value of one; no purple color appeared on their fracture surfaces, indicating the acid had penetrated the whole specimens and led to their neutralization. For the specimens exposed to acetic acid with a pH value of three, the neutralization depths decreased significantly. Among them, the smallest length of one side of the purple square area was 29 mm, that is, the neutralization depth of the AAF mortar was the greatest, i.e., 5.5 mm, followed by the AFS and AAS mortars, which were both about 3.0 mm, and that of OPC mortar was the smallest, i.e., only 1.0 mm. Obviously, the neutralization depths of alkali-activated mortars were greater than that of OPC mortars, moreover, the neutralization depths of alkali-activated mortars decreased with an increase in their slag contents. This was due to the generation of more C-(A)-S-H gels which had finer pores, thus, reducing the penetration of corrosive medium [31]. When they were exposed to sulfuric acid, the neutralization depths of all the specimens were reduced significantly as compared with those in acetic acid with the same pH value. For the specimens in sulfuric acid with a pH value of three, the change rule was similar to that in acetic acid, the neutralization depth of OPC mortar was still the smallest, i.e., 1.5 mm, while that of the AAF mortar was the greatest, i.e., 4.5 mm. When they were exposed to sulfuric acid with a pH value of one, the neutralization depths of the AAS and AFS mortars were still small, however, that of OPC mortar increased greatly due to the spalling of porous corrosion layer. Although the neutralization depth of the AAF mortar was even greater than OPC mortar, its appearance was relatively intact, probably due to the highly connected aluminosilicate network structure.

#### 3.1.4. Strength

Table 3 lists the test results of the flexural strength and compressive strength of different mortars before and after acid corrosion. The initial strengths of AAF and OPC mortars were almost the same, but with the addition of slag in alkali-activated binders, the strength of the mortars more than doubled. 

**Figure 4:**
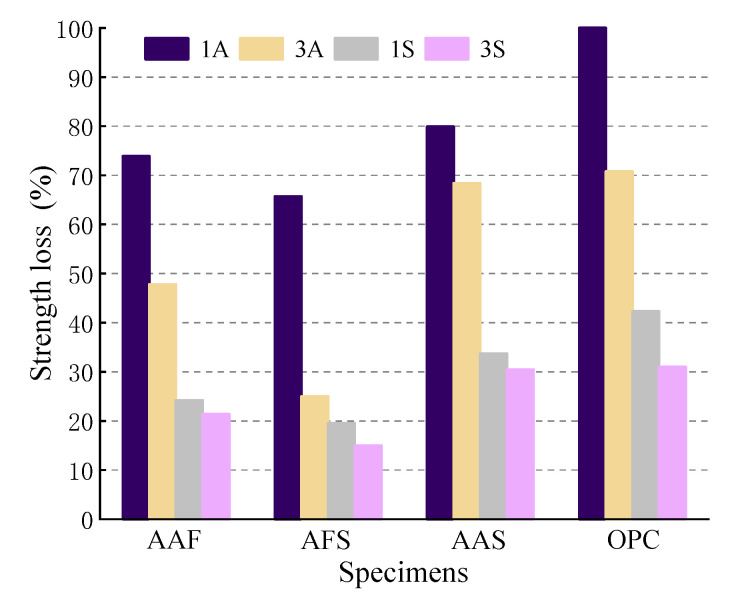
Compressive strength loss of mortar specimens exposed to acid.

After 28 days of immersion in acidic solutions, the strength of all the mortar specimens decreased to varying degrees, especially those exposed to acetic acid, their strength loss all approached or exceeded 50%, except for the AFS mortar, and even reached 100% for the OPC mortar in 1A solution, as shown in Figure 4. For the specimens immersed in sulfuric acid solution, they had much lower strength loss, which may be due to the formation of gypsum in the corrosion layer. In addition, the strength losses of alkali-activated mortars were lower than those of the OPC specimens. Although the initial strength of the AAF mortar was similar to that of the OPC mortar and lower than that of AAS mortar, its strength loss was the lowest. This indicates that a large amount of N-A-S-H gel forms in the AAF mortar, which is less susceptible to acid attack than C-(A)-S-H gel, which is the main product in the OPC and AAS mortars, and the formation of extensive gypsum may cause expansion and high internal stresses when exposed to sulfuric acid. However, the addition of slag in the AAF mortar not only increased the initial strength, but also increased its residual strength significantly. The strength loss of the AFS mortar was the lowest in all of the mortar specimens, especially in sulfuric acid. From the strength degradation results, it can be seen that the acid resistance of mortars from the best to the worst is as follows: AFS > AAF > AAS > OPC.

### 3.2. Observation and Testing of the Changes in Acidic Solutions

#### 3.2.1. Color

**Figure 5:**
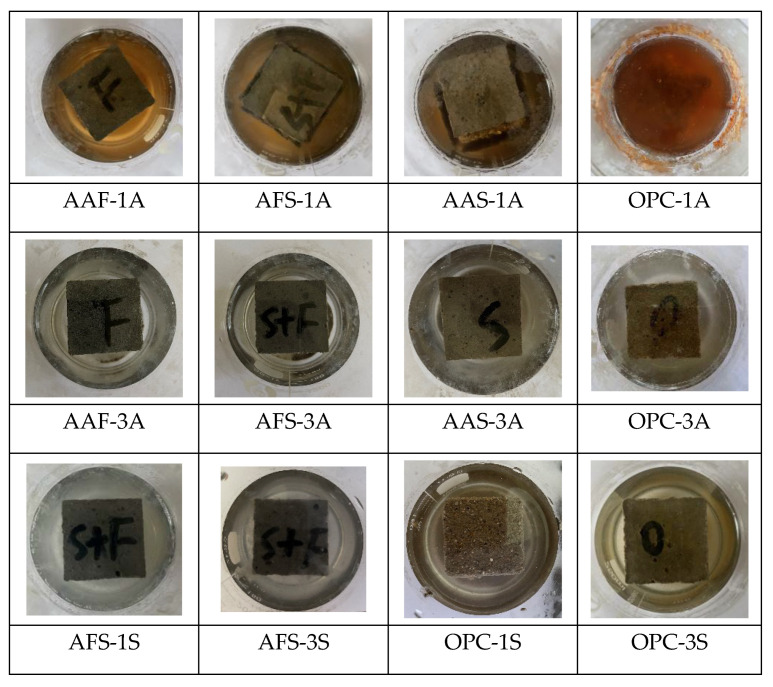
Color change of the acidic solutions.

After being immersed in the acidic solutions, the mortar specimens were observed. Figure 5 shows that, after 7 days of immersion, the acetic solutions with a pH value of one used for immersing each group of mortar exhibited different degrees of red, and among them, the OPC mortar had the deepest color, followed by the AAF mortar. This was because the iron oxide content in cement and fly ash was richer than that in GBFS, and therefore, the leached iron oxide reacted with acetic acid to form red ferric acetate, and further hydrolyzed to generate ferric hydroxide colloid, which was reddish brown and not easy to precipitate. The chemical reaction is as follows:

6CH_3_COOH + Fe_2_O_3_ → 2Fe(CH_3_COO)_3_ + 3H_2_O (1)

Fe(CH_3_COO)_3_ + 3H_2_O → Fe(OH)_3_ + 3CH_3_COOH (2)

When the pH value of the acetic acid solution increased from one to three, all the solutions used for immersing different mortar specimens became obviously lighter in color, but the change in color was similar to that in 1A solution. For the sulfuric acid solutions used for immersing different mortar specimens, there were almost no changes in color, except for OPC-3S, which was pale yellow. Moreover, some white flocculent substance formed around the specimens in sulfuric acid solution, which was inferred to be gypsum. It may have inhibited the leaching of iron, so the color of sulfuric acid solution was lighter.

#### 3.2.2. pH

**Figure 6:**
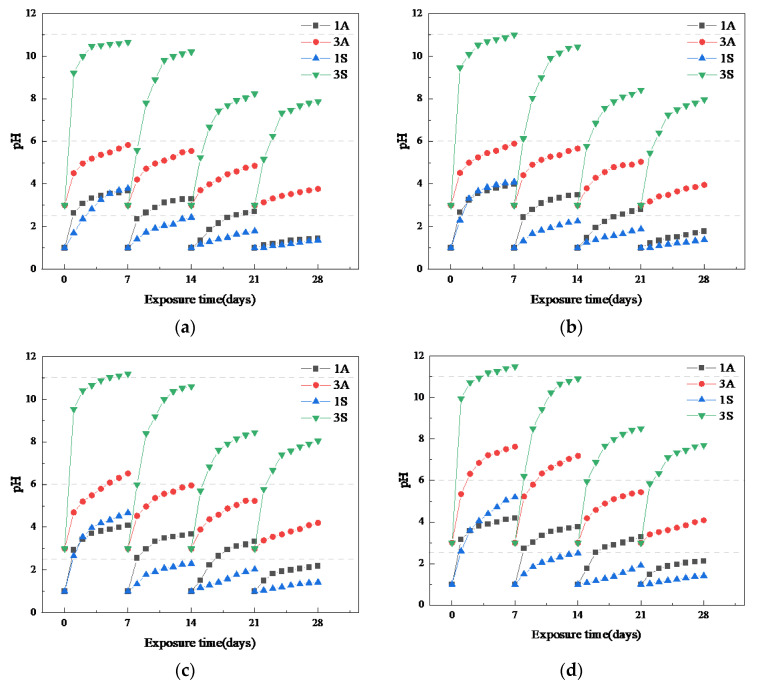
Change of pH values of the acidic solutions with time: (**a**) AAF; (**b**) AFS; (**c**) AAS; (**d**) OPC.

The pH values of the acidic solutions immersed for different mortar specimens were measured every day. The results are shown in Figure 6. It can be seen that the initial pH values of the acidic solutions were controlled around one and three, respectively, but they gradually increased with time, and then fell to the initial value periodically due to the acidic solution supplement every 7 days. For the acidic solutions with a pH value of three, the increases in the pH values of sulfuric acid were significantly higher than those of acetic acid with an increase in immersing time. The pH of the 3S solution had increased to more than 9 after only 1 day, and exceeded 10 after one cycle, and the growth increased with an increase in the calcium content of the sample, even exceeded 11 for that used for immersing the OPC mortar. This resulted from the fast neutralization reaction between alkali solution in pores and acid, and then the dissolution of alkali after the exhaustion of acid. However, with an increase in exposure time, the alkali retained in samples was less and less, and therefore, the pH of the 3S solution gradually decreased and tended to be neutral. Whereas the ionization of acetic acid could supplement the H^+^ consumed in the reaction continuously, and therefore, the pH of the 3A solution increased slowly. On the contrary, for the acidic solutions with a pH value of one, the increase in the pH value of acetic acid was higher than that of sulfuric acid overall. This can be explained as follows: On the one hand, the H^+^ released by ionization could not quickly replenish its consumption due to the harsh reaction that occurred in strong acids; on the other hand, the great amounts of gypsum formed in the 1S solution could act as a barrier to slow down the reaction. Furthermore, the gap between the pH curves of 1A and 1S increased gradually with an increase in the calcium content of the samples. This is due to the dissolution of calcium acetate, the main corrosion product of samples when exposed to acetic acid, which accelerates the penetration and consumption of acid.

### 3.3. Microscopic Analysis of Hardened Paste after Acid Corrosion

#### 3.3.1. SEM-EDS

**Figure 7:**
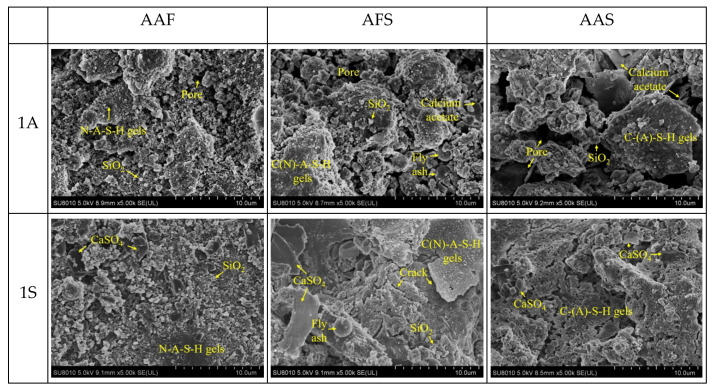
SEM images of alkali-activated materials suffering acetic acid and sulfuric acid attack.

Figure 7 shows the morphology of different alkali-activated mortars after 28 days immersion in solutions of CH_3_COOH and H_2_SO_4_ with a pH value of one. Obviously, the microstructures of specimens suffering sulfuric acid attack were relatively denser than those of specimens exposed to acetic acid. Furthermore, with an increase in the slag content of the mixture, the deterioration of the specimen was more serious. There was relative integrity of the morphology of the AAF mortar specimen after acid erosion, only a small amount of amorphous silica gel and pores could be observed, which was due to the stable three-dimensional network structure of N-A-S-H gel. For specimens exposed to sulfuric acid, the gypsum which formed, further filled the pores, leading to a denser structure. Although the microstructures of the AFS and AAS mortar specimens before corrosion were dense, apparently, they suffered more serious attacks by acidic solutions. For specimens immersed in acetic acid, the structure was porous, and some calcium acetate and SiO_2_ appeared, indicating the dissolution of C-(A)-S-H gel, which had a long chain structure [32] and was vulnerable in the acidic environment. In addition, the structure of the AAS mortar was more porous than that of the AFS mortar. For specimens exposed to sulfuric acid, some gypsum was generated and close packed on the corrosion layer, so the structure was relatively denser. Moreover, the C-A-S-H product contributes to the refinement of the pore network, but it may induce the dry shrinkage, and excessive gypsum would have certain expansive effect, both of them resulted in the formation of many micro-cracks.

**Figure 8:**
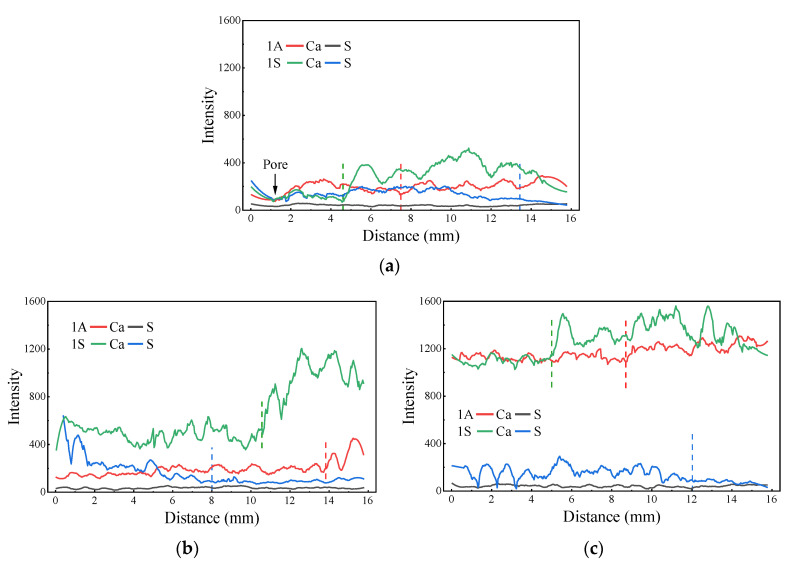
EDS line scanning of different samples suffering acid attack: (**a**) AAF; (**b**) AFS; (**c**) AAS.

Figure 8 shows the line scanning results of the different samples immersed in acetic acid and sulfuric acid solutions, with a pH value of one, over 28 days. The CA and S elements were scanned by EDS. For each sample, the scan range of 16 mm was stitched from four scanning images along the same line. It can be seen that the concentration of Ca in the alkali-activated samples increased from the surface to the interior, indicating penetration of the acid solution, which led to the decalcification of C-(A)-S-H gel and dissolution of calcium. However, the positions of the sharp rise in Ca for samples in acetic acid and sulfuric acid solutions were different, which were indicated by the red and green dashed lines. Obviously, the position of the increase in Ca for samples exposed to acetic acid was further away from the surface as compared with that in sulfuric acid, indicating a deeper acid intrusion. Presumably, the less soluble gypsum remained in the corrosion layer and could resist the permeation of some aggressive acid. Moreover, the concentration of Ca in the samples exposed to acetic acid was lower than that in sulfuric acid. This is due to the higher solubility of calcium acetate as compared with that of the calcium sulfate. They all indicate that acetic acid has a stronger attack effect on the sample structure as compared with sulfuric acid.

Variations in the S element can also indicate penetration of sulfuric acid. From the blue dashed lines in Figure 8, it can be seen that, beyond that position, the content of S became very low, almost close to that of the samples in acetic acid, meaning that acid did not infiltrate into this area. Obviously, the penetration depth of the AFS mortar was less than that of the AAF and AAS mortars. Since the AAF mortar was dominated by more porous N-A-S-H gel, the penetration depth of acid was greater. The cracks that occurred in the corrosion layer of the AAS mortar, which were caused by the formation of excessive gypsum, also increased the diffusion of acid. In addition, S element content in the area of 0–2 mm of the AFS mortar was relatively higher, which was probably derived from gypsum in the corrosion layer. This was not observed in the AAS mortar, which possibly resulted from the spalling of the loose corrosion layer.

#### 3.3.2. XRD

**Figure 9:**
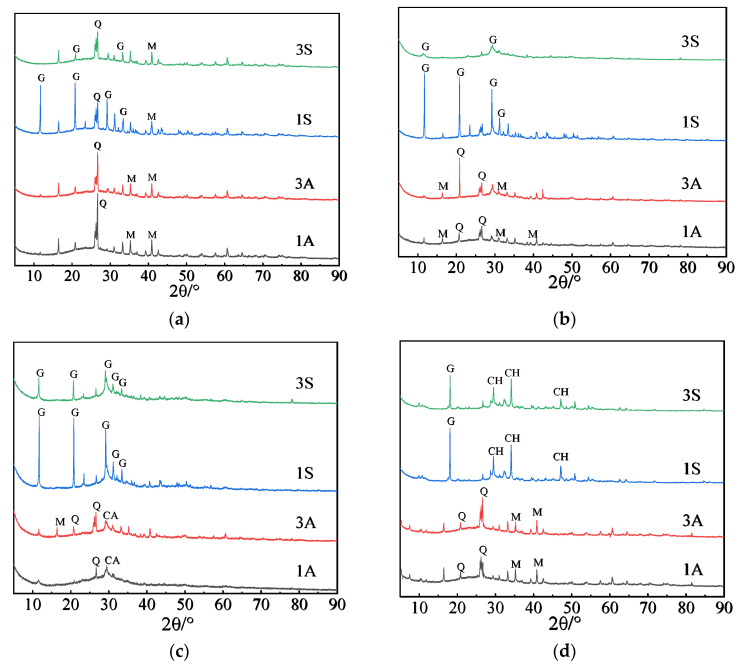
XRD patterns of samples after immersion in acidic solutions for 28 days (G, CaSO_4_; Q, SiO_2_; M, mullite; CH, Ca(OH)_2_; CA, calcium acetate): (**a**) AAF; (**b**) AFS; (**c**) AAS; (**d**) OPC.

The mineral compositions of the corrosion layers of different samples after 28 days of exposure to acid solutions were analyzed and the results are shown in Figure 9. For AAF mortar, the main peaks were mainly related to quartz and mullite, indicating that the N-A-S-H gel product was decomposed in the acetic acid environment. In addition, the quartz’s peak intensity in the AAF mortar exposed to acetic acid was higher and slightly wider as compared with that suffering sulfuric acid attack. This is possibly due to the ionization of acetic acid, which can replenish the consumption of H^+^ continuously. Thus, it had a stronger attack effect on the AAF mortar, resulting in the damage of cross-linked aluminosilicate skeleton and formation of finer SiO_2_. When the AAF mortar was exposed to sulfuric acid, the peak of gypsum could also be observed, and its intensity increased as the acid concentration increased (Figure 9a). This is due to the reaction of a small amount of calcium in fly ash with sulfuric acid. For the AFS mortar, the intensity of the SiO_2_ peak significantly decreased and that of the gypsum peak obviously increased as the slag content was increased in binders (Figure 9b). This was because, when the pH of solution was lower than two, H^+^ from acid ionization attacked the aluminosilicate network, and then led to the leaching of calcium ions. However, for the AAS mortar, a very prominent peak of gypsum could be detected and even suffered 3S solution attack with a low concentration. Moreover, the peak of calcium acetate was observed at 29° 2θ after exposure to acetic acid, as well as some SiO_2_ phase (Figure 9c). This indicated the decalcification of the C-(A)-S-H gel in the sample was caused by the acid attack. For the OPC mortar, the peaks of Ca(OH)_2_ and gypsum could be observed after exposure to sulfuric acid, whereas there was no obvious Ca(OH)_2_ peak in the acetic acid environment, and only some quartz and mullite were formed, due to the dissociation of hydration products, indicating more severe acid attack (Figure 9d).

#### 3.3.3. AC Impedance Spectra

**Figure 10:**
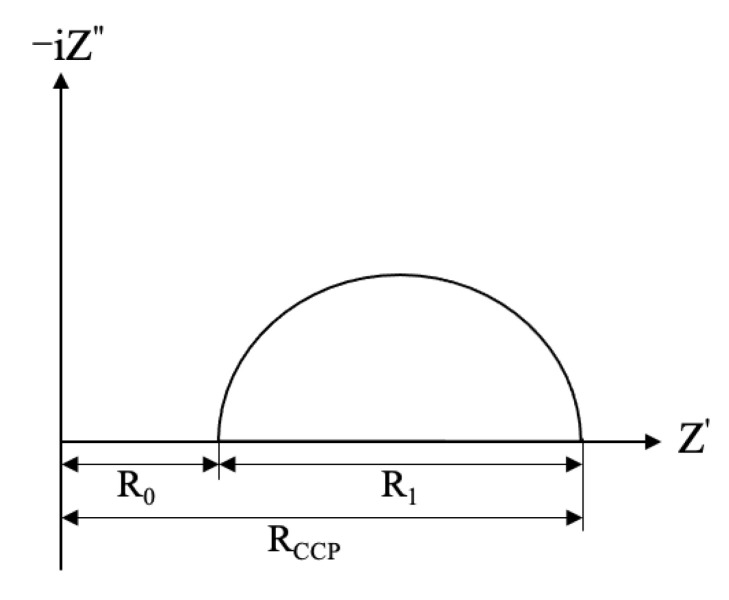
Typical Nyquist plot for concrete. Adapted with permission from ref. [34]. 2000 Elsevier.

AC impedance spectroscopy is a nondestructive method that can be used to characterize the microstructure features in cement-based materials [33]. In the equivalent circuit model proposed by Song [34], the resistance of all continuously and discontinuously connected micropores that filled with water in concrete was described as *R*_0_, which was inversely proportional to the porosity, and the resistance of the continuously connected micropores was described as *R*_CCP_. A typical Nyquist plot for concrete is shown in Figure 10, and the reaction kinetic parameter *R*_1_ can be obtained from the diameter of a high-frequency semicircle.

**Figure 11:**
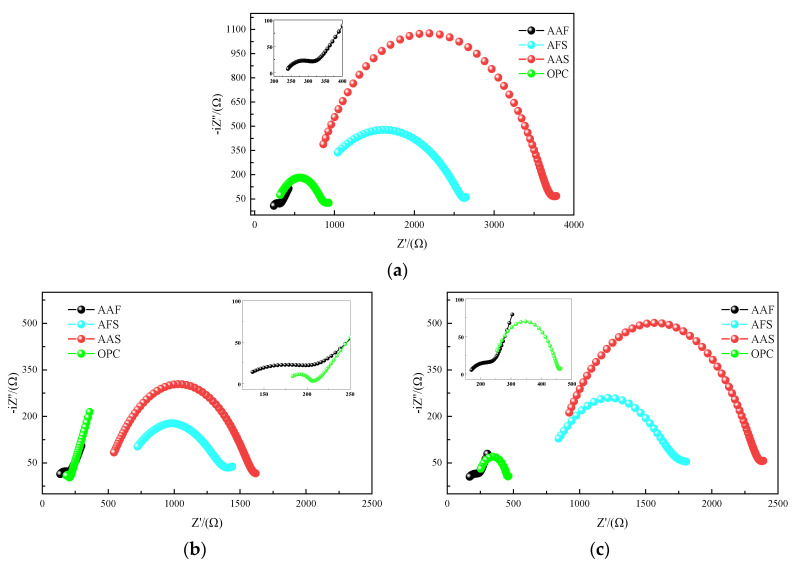
Nyquist diagrams of AC impedance test: (**a**) Before corrosion; (**b**) after 14 days corrosion in 3A solution; (**c**) after 14 days corrosion in 3S solution.

Figure 11 displays the AC impedance spectra of different samples. The shapes of the Nyquist diagram varied greatly before corrosion, as shown in Figure 11a. The high-frequency semicircle of the AAF mortar was obviously smaller than that of the other samples, see the enlarge view. With an increase in slag content of alkali-activated binders, the diameter of high-frequency semicircle increased significantly, and the position moved to the right gradually. Moreover, the equivalent circuit model parameters (*R*_0_, *R*_1_) were extracted from the impedance spectra using Zview software, and the results are listed in Table 4. It can be seen that the parameters *R*_0_, *R*_1_, and *R*_CCP_ of the AAF mortar were 229 Ω, 137 Ω, and 366 Ω, respectively, indicating the low activity of fly ash and insufficient alkali-activated reaction, thus, there were many connected micropores in it. However, the parameters of both the AFS and AAS mortars increased sharply, and the *R*_CCP_ of AAS mortar was as high as 3883 Ω, almost 10 times that of the AAF mortar. This was due to the addition of slag which resulted in the formation of denser C-(A)-S-H gels in alkali-activated mortar, which acted by filling the space and reducing the porosity. In addition, the high-frequency semicircle of OPC mortar laid between the AAF and AFS mortars, and it had similar *R*_0_ and greater *R*_1_ as compared with the AAF mortar, indicating that, although their porosity was almost the same, the OPC mortar had less connected pores than the AAF mortar.

Figure 11b,c shows the Nyquist diagrams of different samples exposed to 3A and 3S acidic solutions for 14 days. It can be seen that the diameters of high-frequency semicircles of all the samples decreased as compared with those of the samples before corrosion. If the samples were cured for 14 days, the impedance parameters showed some increase with the continuous reactions. Obviously, they all suffered acid attack. Furthermore, the positions of the Nyquist curves move to the left, especially for the samples in the 3A solution, indicating a decrease in *R*_0_, in other words, an increase in the porosity. This indicates that the acetic acid is more aggressive on samples than sulfuric acid, which is consistent with the above results.

**Figure 12:**
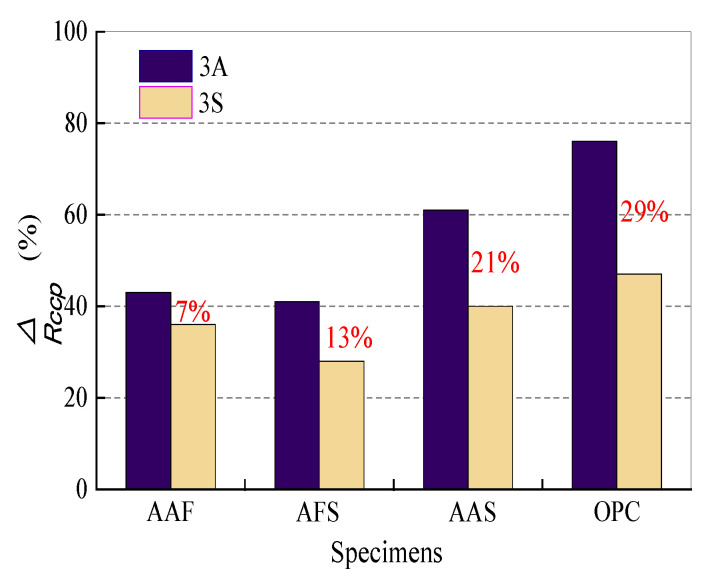
Comparison of the impedance change of samples after 14 days of immersion in acetic acid and sulfuric acid solutions with a pH value of 3.

In order to further compare changes in sample microstructures, the differences in *R*_CCP_ values before and after corrosion were calculated and are listed in Table 4. In the acetic acid environment, the Δ*R_CCP_* values of the OPC mortar are the largest, followed by AAS, AAF, and AFS mortars. The same rule also existed in the sulfuric acid environment, but, in general, the Δ*R_CCP_* values were smaller. Taking into consideration all the findings above, the deterioration of samples in the acetic acid solution is characterized by decomposition and dissolution of the products, resulting in an increase in porosity and a decrease in impedance. However, in sulfuric acid solution, some gypsum is generated in the corrosion layer, which can fill the pores and decrease the penetration of acid, but excessive gypsum may cause expansion. Figure 12 compares the Δ*R_CCP_* values of different samples exposed to 3A and 3S solutions; the Δ*R_CCP_* values were 7%, 13%, 21%, and 29% for AAF, AFS, AAS, and OPC mortars, respectively. It is noticeable that the gap in the impedance change between samples immersed in acetic acid and sulfuric acid solutions increased with an increase in the calcium content of binders; this may lead to the formation of more gypsum in the corrosion layer when suffering sulfuric acid attack. Therefore, it can be inferred that an appropriate amount of gypsum existed in the corrosion layer which acted as a barrier to some extent in the sulfuric acid with a pH of three. The AC impedance test of samples exposed to acidic solution with a pH of one was not carried out due to the serious spalling of the surface layers. Perhaps the adverse effect of expansive gypsum on the acid resistance of samples should be considered in this environment. Briefly, an increase in the calcium contents of binders led to the generation of growing amounts of unstable C-(A)-S-H gels in samples, which reduced their resistance to acid attack; however, the formation of gypsum in sulfuric acid could mitigate the corrosion due to its pore filling effect.

## 4. Conclusions

1. When exposed to an acetic acid environment, OPC mortar experienced more severe deterioration in terms of appearance, mass loss, and strength loss as compared with alkali-activated mortars, but its neutralization depths were smaller, except for that in the 1S solution, resulting from the spalling of gypsum corrosion layer. The AAF mortar had a relatively intact appearance but the largest neutralization depth, which was due to its stable three-dimensional network but highly porous structure. The AC impedance results showed that, although the initial porosity of the AAF mortar was similar with that of the OPC mortar, the former had more connected pores; however, with increased slag in the binders, the parameter *R*_CCP_ increased sharply, indicating the formation of denser structure. To sum up, AFS mortar has the best resistance to acid attack.
2. By comparing the performance of mortar specimens in both acetic and sulfuric acids with the same pH value, it was found that the mortars in acetic acid suffered greater deterioration, which was mainly governed by the products and structure of the corrosion layer. The dealumination or decalcification of N-A-S-H/C-(A)-S-H gels and dissolution of calcium acetate led to the formation of a highly porous corrosion layer in acetic acid, whereas the crystallization of gypsum within the corrosion region when exposed to sulfuric acid had a pore filling effect. The color and pH changes of the acidic solution indicated that the gypsum formed could inhibit the leaching of iron and slow down the neutralization reaction between alkali solution in pores and acid. However, for AAS and OPC mortars exposed to sulfuric acid, extensive gypsum resulted in the formation of micro-cracks, and the corrosion layer of the OPC mortar was more prone to fall off.
3. In the acetic acid environment, the OPC mortar had the largest Δ*R_CCP_* value, which is the resistance difference of the continuously connected micropores before and after corrosion, followed by AAS, AAF, and AFS mortars, and the Δ*R_CCP_* values for all the specimens were smaller in sulfuric acid. Furthermore, the Δ*R_CCP_* values of different samples exposed to acetic and sulfuric acid solutions were compared, and the gap values increased with an increase in the calcium content of binders, which were 7%, 13%, 21%, and 29% for AAF, AFS, AAS and OPC mortars, respectively. Thus, it can be inferred that an appropriate amount of gypsum existing in the corrosion layer can act as a barrier to some extent in the sulfuric acid with a pH of three. Perhaps the adverse effect of expansive gypsum on the acid resistance of samples should also be considered in sulfuric acid with a pH of one.

## Figures and Tables

**Table 1:** Chemical composition of fly ash, GBFS, and cement as determined by XRF.

Composition wt%	CaO	SiO_2_	Al_2_O_3_	MgO	Fe_2_O_3_	P_2_O_5_	TiO_2_	K_2_O	Na_2_O	SO_3_	Loss
Fly ash	2.82	58.30	30.06	0.17	2.68	0.38	1.07	1.84	1.13	0.61	3.79
GBFS	38.70	30.21	14.42	9.33	0.46	-	1.82	0.15	0.51	2.10	2.30
Cement	62.40	21.35	4.76	3.06	3.13	0.49	-	0.21	0.54	2.52	1.54

**Table 2:** Mixture proportions of mortar (kg/m^3^).

Ingredients	Fly Ash	Slay	Cement	Sand	Added Water	NaOH (10 M)	Na_2_SiO_3_ (Liquid)
AAF	450	-	-	1350	45	70	200
AFS	225	225	-	1350	45	70	200
AAS	-	450	-	1350	45	70	200
OPC	-	-	450	1350	225	-	-

**Table 3:** Strength degradation of the mortars exposed to acidic solutions.

No.	Compressive Strength (MPa)		Strength Loss (%)	Flexural Strength (MPa)		Strength Loss (%)
	Before	After		Before	After	
AAF-1A	32.2	8.4	73.9	5.35	1.67	69.0
AAF-3A		16.8	47.8		2.55	52.3
AAF-1S		24.4	24.2		3.95	26.2
AAF-3S		25.3	21.4		4.32	19.3
AFS-1A	78.1	26.8	65.7	11.75	5.52	53
AFS-3A		58.6	25.0		8.75	25.5
AFS-1S		62.9	19.5		9.13	22.3
AFS-3S		66.4	15.0		9.55	18.7
AAS-1A	65.8	13.2	79.9	12.00	3.21	73.0
AAS-3A		20.8	68.4		5.08	57.7
AAS-1S		43.6	33.7		7.67	36.1
AAS-3S		45.8	30.4		8.65	27.9
OPC-1A	33.6	-	100.0	5.71	-	100
OPC-3A		9.8	70.8		2.18	61.8
OPC-1S		19.4	42.3		3.45	39.6
OPC-3S		23.2	31.0		3.95	30.8

**Table 4:** Impedance parameters of the samples.

Impedance Parameter/Ω	Before			3A				3S			
	*R*_0_	*R*_1_	*R*_CCP_	*R*_0_	*R*_1_	*R*_CCP_	Δ*R*_CCP_ (%)	*R*_0_	*R*_1_	*R*_CCP_	Δ*R*_CCP_ (%)
AAF	229	137	366	125	83	208	43	165	69	234	36
AFS	701	1728	2429	599	822	1421	41	649	1112	1761	28
AAS	918	2965	3883	508	1011	1519	61	823	1492	2315	40
OPC	260	581	841	177	28	205	76	234	214	448	47
